# Interdental brushes for improving oral hygiene in patients with fixed orthodontic appliances: a systematic review

**DOI:** 10.3389/froh.2026.1707988

**Published:** 2026-05-04

**Authors:** Xinyue Ji, Qiao Zou, Lei Tian

**Affiliations:** 1 School of International Pharmaceutical Business, China Pharmaceutical University, Nanjing, China; 2 Center for Pharmacoeconomics and Outcomes Research, China Pharmaceutical University, Nanjing, China

**Keywords:** interdental brush, oral hygiene, orthodontics, plaque control, systematic review

## Abstract

**Background:**

An interdental brush (IDB) is an adjunctive oral-hygiene tool designed to clean interproximal spaces and remove dental plaque. This study systematically evaluated its effectiveness for improving oral hygiene and periodontal health among orthodontic patients.

**Methods:**

A systematic search was conducted across five databases including PubMed, EMBASE, Cochrane, Web of Science, Scopus up to 30 August 2025. Randomized controlled trials enrolling orthodontic patients that compared IDB with other adjunctive oral-hygiene aids or with no adjunctive were included. The primary endpoint was periodontal condition, measured by periodontal indices. We assessed risk of bias with the Cochrane risk of bias tool. Given clinical and methodological heterogeneity, findings were synthesized narratively following SWiM guidance. The certainty of evidence for primary outcomes was evaluated using the GRADE framework.

**Results:**

Six studies with a total of 442 orthodontic patients were included in the systematic review. Low-certainty evidence from individual studies suggested that IDB may reduce plaque and gingival inflammation compared with toothbrushing alone or floss. Greater benefits were observed when IDBs were combined with orthodontic toothbrushes, whereas no consistent advantage was demonstrated over monotufted brushes or oral irrigators. Outcome measures and indices varied substantially across trials, follow-up periods were generally short, and safety outcomes were rarely reported. These limitations restricted the ability to pool results and lowered the certainty of evidence.

**Conclusion:**

Low-certainty evidence from a limited number of heterogeneous trials provides only tentative indications that interdental brushes may help improve gingival health and plaque control in orthodontic patients—particularly when used with orthodontic toothbrushes. High-quality, multicenter trials with standardized protocols and longer follow-up are needed to guide clinical recommendations.

**Systematic Review Registration:**

https://www.crd.york.ac.uk/PROSPERO/view/CRD420251136657, identifier CRD420251136657.

## 1 Introduction

Orthodontic treatment corrects malocclusion and dentofacial discrepancies, ultimately improving function, aesthetics, and quality of life (1). During treatment, however, fixed appliances such as brackets, archwires, bands, and attachments tend to create additional plaque-retentive sites and complicate oral hygiene. Through increased surface irregularities and niche formation, these devices are associated with biofilm accumulation and shifts in the subgingival microbiota toward periodontal pathogens, contributing to gingivitis, white-spot lesions, and caries (2, 3).

Given these risks, daily mechanical plaque control is particularly crucial for patients with fixed appliances. While toothbrushing is the most common way of plaque removal, it fails to reach interdental spaces, and is less effective on proximal tooth surfaces compared to buccal and lingual surfaces (4). Evidence shows that 20%–40% of fixed orthodontic patients exhibit suboptimal plaque control when relying on toothbrushing only (5, 6). Adjunctive cleaning with interdental tools is therefore recommended for plaque control (7). Common options include dental floss, oral irrigators, and interdental brushes (IDB), which help remove plaque from proximal surfaces.

Although dental floss is recommended for the prevention of periodontal disease and dental caries in both children and adults (8), adherence is generally low (9, 10) and use is particularly challenging for patients with fixed orthodontic appliances. Oral irrigators may offer advantages for gingival protection (11) and patient adherence, yet current evidence in orthodontic populations shows they do not significantly improve oral hygiene (12). An interdental brush typically features a small head of soft nylon filaments arranged perpendicularly around a central wire core, enabling access to interproximal spaces that are difficult to clean with toothbrushing alone. In non-orthodontic populations, a network meta-analysis comparing ten interdental cleaning aids suggested that, as an adjunct to toothbrushing, IDB achieve the greatest reductions in gingival index (13). Several recent clinical studies have also demonstrated that routine use of IDB reduces interproximal gingival bleeding (14–16). To date, however, the impact of IDB on orthodontic patients remains unclear. This study aims to synthesize the existing literature to evaluate the effects of IDB on oral hygiene and periodontal health in orthodontic populations.

## 2 Methods

This systematic review adhered to the Preferred Reporting Items for Systematic Reviews and Meta-Analyses (PRISMA 2020) statement guidelines and followed Cochrane Handbook for Systematic Reviews of Interventions. The protocol has been registered in PROSPERO: CRD420251136657.

### 2.1 Search strategy

A computerized search was conducted through the following electronic databases: PubMed, EMBASE, Cochrane, Web of Science, Scopus, covering records from their inception to 30 August, 2025. Complete search strategies of each database are available in Supplementary Table S1. Additional searches were conducted based on published systematic reviews and the “Cited by” records of the retrieved full-text articles. No language restrictions were applied.

### 2.2 Eligibility criteria

The inclusion criteria were formulated in accordance with PICOS: (a) Population: patients undergoing fixed orthodontic appliance; (b) Intervention: adjunctive use of interdental brushes to toothbrushing; (c) Comparison: toothbrushing alone or combined with adjunctive aids; (d) Outcome: Indices related to periodontal health status. (e) Study: Randomized clinical trials (RCTs) including parallel-group, crossover, or split-mouth designs.

The following literature was excluded: systematic reviews, animal studies, and other non-RCTs; studies involving participants with severe periodontal diseases; studies with unavailable full texts or results.

### 2.3 Literature selection and data extraction

Two authors (X.J and Q.Z) independently screened the titles and abstracts of all records identified through database and manual searches. Duplicate records were removed prior to screening using both reference manager tools and manual verification. Full texts were retrieved and evaluated when the relevance could not be ascertained based on titles and abstracts alone. Any discrepancies were resolved through discussion with a third reviewer (L.T). Reviewers were not blinded to study authorship, institution, or journal source.

All studies deemed eligible underwent data extraction. The same two authors independently extracted data using a standardized data extraction form. Disagreements were resolved through discussion or adjudicated by a third reviewer when necessary. Inter-rater reliability was assessed using Cohen's kappa coefficient, with a score of 0.85 indicating excellent agreement between the two reviewers during study selection. The following information was extracted from the included studies: study design, sample size, baseline characteristics of participants, type and brand of cleaning tools, comparator group, anticaries or fluoride-containing products in protocol, timing of outcome assessments, primary outcomes, and main findings.

### 2.4 Risk of bias evaluation

The methodological quality of included studies was independently assessed in duplicate by two reviewers (X.J and Q.Z) using the Cochrane Risk of Bias Assessment Tool. Each domain was rated as having a low, high, or unclear risk of bias. A study was considered to have an overall low risk of bias only if all domains were rated as low. If any domain was rated as high risk, the study was classified as high risk of bias; if one or more domains were rated unclear with no high-risk judgments, the study was categorized as having an unclear overall risk.

### 2.5 Evidence grading

The primary outcomes were assessed according to the Grading of Recommendations Assessment, Development, and Evaluation (GRADE) system, which evaluates evidence quality based on risk of bias, imprecision, inconsistency, and indirectness. Evidence was classified as follows: high quality (no downgrades), moderate quality (downgraded by 1 level), low quality (downgraded by 2 levels), or very low quality (downgraded by 3 levels).

### 2.6 Data synthesis

Given the heterogeneity in study design, interventions, and outcome measures, quantitative meta-analysis was not feasible. Therefore, a structured narrative synthesis was undertaken. Following SWiM guidance (17), studies were grouped by comparator. Comparator groups were: (i) toothbrushing plus IDB vs. toothbrushing alone, and (ii) toothbrushing plus IDB vs. toothbrushing plus other adjunctive interdental aids. All effects were harmonized so that lower scores indicate better outcomes. We reported study-level mean differences (MD) with 95% confidence intervals (CI) in original units for outcomes measured on the same or comparable scales, and expressed percentage outcomes as percentage-point MD. Otherwise, direction only was summarized. Heterogeneity was explored descriptively through subgroup comparisons.

## 3 Results

### 3.1 Search results and study selection

**Figure 1:**
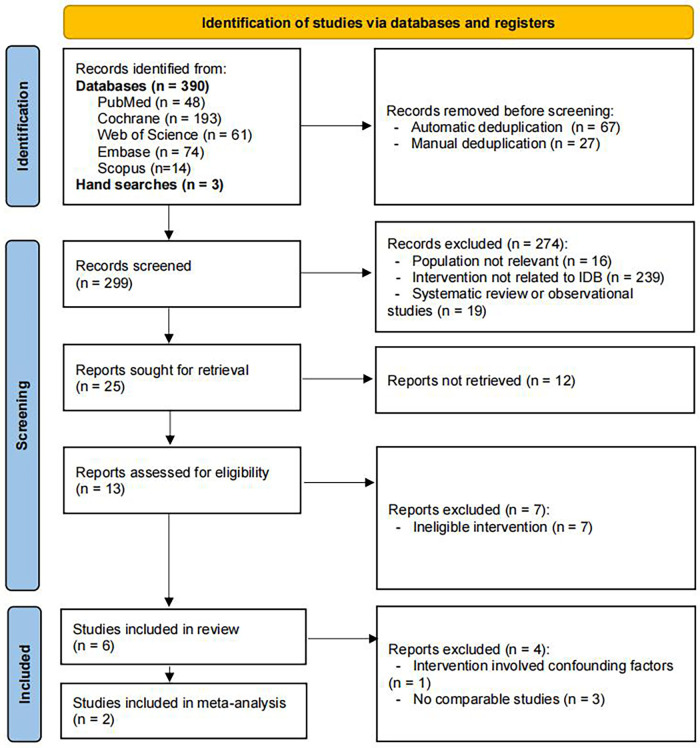
Flow diagram of literature selection.

A total of 390 records were identified through database searches, and an additional 3 studies were retrieved via manual searching. After removing duplicates, 299 records remained. Following title and abstract screening, 25 studies were considered potentially eligible. However, full-text articles were available for only 13 of them. Of the full-text studies assessed, 7 were excluded due to non-eligible interventions. More specific reasons for exclusion are provided in Supplementary Table S2. Ultimately, 6 studies met the inclusion criteria (18–23). The process of literature search and selection was described in Figure 1.

### 3.2 Characteristics of included studies

The characteristics of the included studies are summarized in Table 1.

**Table 1:** Characteristics of included studies.

Authors	Year	Study design	Sample size (trial/control arm)	Gender	Mean age/Range	Comparison	Brand of cleaning tools	Anticaries or fluoride-containing products in protocol	Observation time	Outcome	Conclusion
Arici et al. (18)	2007	3-regimen crossover, assessor-blinded trial	30	12 M 18 F	13–16	- Group1: curved-bristle toothbrush (CBT) - Group2: orthodontic toothbrush (OT) - Group3: orthodontic toothbrush + interproximal toothbrush (OT + IT)	- CBT (Collis-Curve Inc.) - OT, IT (Oral-B Laboratories, Inc.)	Standard fluoride toothpaste provided; use of any other oral agents (mouthwash, irrigators) was prohibited	20 weeks total: baseline, 4 weeks (CBT), 12 weeks (OT), 20 weeks (OT + IT); 4-week washout interval between each protocol	- Gingival Index (GI; WHO, 1978) - Percentage Plaque Index (PPI) - Percentage Interproximal Plaque Index (PIPI)	- The use of CBT and OT alone was limited in effectively removing plaque beneath the archwires. - OT + IT protocol significantly reduced PPI, PIPI, and GI.
Bock et al. (19)	2010	prospective, randomized, observer-blind, crossover, split-mouth clinical trial	104	39 M 65 F	13.5 (F), 13.7 (M); Range: 11–17	- Group1: long straight handle, monotufted head toothbrush + manual toothbrush - Group2: IDB + manual toothbrush	- IDB (elmex® No. 6) - monotufted head toothbrush (TePe® Compact Tuft) - manual toothbrush (elmex® InterX short head)	Anticaries toothpaste, anticaries mouthrinse provided; no other products allowed	24 weeks total: baseline (T0), 6 weeks (T1), 12 weeks/crossover (T2), 18 weeks (T3), 24 weeks (T4); professional dental cleaning was performed to standardize the baseline at T0/T2	- Plaque Index (PI; Attin, 2005) - Self-reported handling experience using Visual Analogue Scale (VAS)	- Both IDB and monotufted head toothbrush significantly reduced plaque index, with no significant difference in PI between brushes. - IDB was preferred by patients due to less pain, easier handling, and better perceived access behind archwires.
Zingler et al. (20)	2014	single-center, stratified, 4-arm parallel, randomized, observer-blind clinical trial	118 (28/30/29/31)	55 M 63 F	Median 13.0 years (IQR 12.25–13.9); Range: 11–15	- Group 1: sonic toothbrush + sealant (STB + SS) - Group 2: manual toothbrush + IDB + sealant (MTB + IDB + SS) - Group 3: manual toothbrush + sealant (MTB + SS) - Group 4: manual toothbrush (MTB)	- IDB (Curaprox® CPS 15 interdental brush with UHS 410 handle) - STB (Sonicare® FlexCare with ProResults brush head) - MTB (elmex® interX short brush-head toothbrush)	Not reported	12 weeks total: baseline, 4 weeks, 8 weeks, 12 weeks	- Plaque index around brackets (PIB; Trimpeneers 1997, Clerehugh 1998) - TQHI (Quigley and Hein, 1962; Turesky, 1970) - Modified Approximal Plaque Index (MAPI; Zimmer, 2005) - The decayed, missing, and filled teeth/surface (DMFT/DMFS; WHO, 1997)	- Across the three cleaning strategies (STB + SS vs. MTB + IDB + SS vs. MTB + SS), there were no significant differences in plaque or gingival indices. - MTB + IDB arm showed longer brushing time but no superior clinical outcomes. - The sealant did not significantly affect buccal plaque/gingival measures.
Umalkar et al. (21)	2023	prospective interventional, 2-arm parallel, single-center study	100 (50/50)	- IDB group: 26 M, 24 F - Floss group: 25 M, 25 F	15–30	- Group1: IDB + toothbrushing (TB) - Group2: interdental floss + toothbrushing	IDB (Thermoseal Proxa NS)	Not reported	3 months total: baseline and 3 months	- Gingival Index (GI; Löe, 1967) - Plaque Index (PI; Löe, 1967)	- Both groups improved vs. baseline. - IDB outperformed floss, achieving greater reductions in PI and GI (*p* < 0.01).
Anupama et al. (22)	2018	single-center, investigator-blinded, parallel-group randomized controlled clinical trial	60 (30/30)	Not reported	Not reported	- Group1: orthodontic toothbrush only (OT) - Group2: orthodontic toothbrush + interdental brush (OT + IDB)	- OT (STIM® 42) - IDB (STIM® PROXA – Extra fine, Global Dent Aids)	Colgate® toothpaste provided; not to use any additional oral-hygiene aids	4 weeks total: baseline (day 0), 1 week, 2 weeks, 4 weeks; preceded by a 4-week maintenance/supervision run-in.	- Bonded Bracket Plaque Index (BBPI; Kilicoglu, 2012) - Gingival Index (GI; Loe and Sillness, 1967) - Gingival Bleeding Index (GBI; Ainamo and Bay)	OT + IDB was superior to OT alone in controlling plaque, gingival inflammation, and bleeding.
Esma et al. (23)	2022	Randomized controlled clinical trial	30 (15/15)	Not reported	12–18	- Group1: oral irrigator (OI) + manual toothbrushing - Group2: IDB + manual toothbrushing	Oral irrigator (Aquapick AQ-300)	No non-study mouthwash allowed	8 weeks total: baseline, week 2, week 4, week 8	- Plaque Index (PI; Silness and Löe, 1964) - Gingival Index (GI; Silness and Löe, 1967) - Bleeding on Probing (BOP) - Probing Pocket Depth (PPD) - Clinical Attachment Level (CAL) - Biochemical Evaluation: inter- leukin (IL)-1β, IL-10, matrix metallopro- teinase (MMP)-1, MMP-8 mediators	- Compared with the IDB group, the OI group had lower PI, GI, and BOP. - No significant between-group differences were detected for IL-10 or MMP-1.

#### 3.2.1 Study design and setting

Among the six included RCTs, published between 2007 and 2023, three were 2-arm parallel trials (21–23) and one was a 4-arm trial (20). One study employed a crossover design (18), while another incorporated both crossover and split-mouth designs (19). All included trials were conducted in university or hospital settings, with Zingler's study additionally involving private orthodontic practices (20).

#### 3.2.2 Target population

A total of 442 orthodontic patients with fixed appliances were included across 6 RCTs. Age and gender distributions were reported in 4 studies (18–21). One study did not report age or gender data (23), and another lacked gender information (22). Based on the available data, participants' ages ranged from 11 to 30 years, with 157 males and 195 females. One study reported a significant difference in age distribution between groups: participants in the IDB group were primarily aged 15–20 years, while those in the floss group were mostly aged 21–30 years (21). Regarding baseline periodontal status, four studies included participants with clinically healthy periodontal conditions and performed professional prophylaxis prior to the intervention (19, 20, 22, 23). In one study, patients were eligible if at least 10% of tooth surfaces exhibited plaque (18). Another study reported that baseline levels of plaque and gingival inflammation were notably high among participants (21).

Because each primary comparison included a different subset of trials, baseline characteristics were additionally stratified by main interventions to aid interpretation. Detailed sample composition, age, orthodontic appliance type, and available treatment-duration information for each comparison are summarized in Table 2.

**Table 2:** Characteristics of participants stratified by intervention.

Author/Year	Mean age/Range	Type of orthodontic therapy	Stage of orthodontic therapy at baseline
Toothbrushing plus interdental brush versus toothbrushing			
Arici et al. (18)	13–16	Fixed labial appliances (stainless steel brackets, Tip-Edge®)	The second stage of orthodontic treatment (after levelling and alignment)
Zingler et al. (20)	Median age in years (IQR): TB + IDB + SS: 12.9 (12.2–13.7); TB + SS: 13.0 (12.3–14.4)	Fixed labial appliances (stainless steel brackets, Victory™ Low Profile, 0.022 inch slot, 3M ESPE AG, Seefeld, Germany)	The first 3 months of treatment
Anupama et al. (22)	Not reported	Fixed labial appliances (Begg's appliance)	Active fixed orthodontic treatment
Interdental brush versus other interdental adjuncts			
Bock et al. (19)	13.5 (females), 13.7 (males); Range: 11–17	Fixed labial appliances (Tip-Edge®, TP Orthodontics Inc., La Porte, Indiana, USA)	After 6 months of treatment
Umalkar et al. (21)	15–30	Fixed labial appliances	Not reported
Esma et al. (23)	12–18	Fixed labial appliances	The initial stage of orthodontic treatment

TB, toothbrushing; SS, sealant.

##### 3.2.2.1 Toothbrushing plus interdental brush vs. toothbrushing

Three trials contributed to this comparison (18, 20, 22), involving a total of 149 participants. One crossover trial contributed 30 participants to both the intervention and control arms (18), while the remaining parallel-group trials contributed 60 participants to the intervention arm and 59 to the control arm (20, 22). All participants were treated with fixed labial appliances. Reported ages ranged from 12.2 to 16 years. At baseline, patients in one study were at the second stage of orthodontic treatment after leveling and alignment (18), in another study were in the first 3 months of treatment (20). One trial did not report orthodontic treatment duration (22).

##### 3.2.2.2 Interdental brush vs. other interdental adjuncts

Three trials contributed to this comparison, with 234 participants (19, 21, 23). A total of 104 patients came from a split-mouth crossover trial, while the remaining patients were from a parallel controlled trial, with 65 patients in both the intervention and control arms. The bracket type was uniformly labial fixed brackets, and the age range of participants was 11–30 years. At baseline, patients in one study had already undergone 6 months of orthodontic treatment (19), in another study were about to start orthodontic treatment (21), and in a third study, this information was not reported (23).

#### 3.2.3 Intervention

All included studies featured at least one intervention arm combining toothbrushing with IDB, although the type of toothbrush varied. Specifically, two studies employed orthodontic toothbrushes (OT) in the IDB arm (18, 22), while the remaining four used manual toothbrushes (MTB) (19–21, 23). In one study, a sealant was also applied as part of the intervention protocol (20).

Except for one study (23), the brand of the IDB was reported. Additionally, four studies reported the brand of the toothbrush (18–20, 22). Furthermore, four studies specified the use of anticaries or fluoride-containing products (18, 19, 22, 23). In two of these studies, participants were provided with standardized fluoride toothpaste and instructed to avoid using any other oral hygiene products, including mouthwash or oral irrigators (18, 22). One study supplied both anticaries toothpaste and mouthwash and other oral products were not allowed (19). Another study did not permit the use of non-study mouthwash (23).

#### 3.2.4 Outcome measures

Outcome assessments were conducted at baseline and multiple follow-up points in all studies, though follow-up durations varied substantially, ranging from 4 weeks to 24 weeks. A broad spectrum of clinical outcomes was reported across the included studies, particularly focusing on plaque and gingival status.

For plaque assessment, common whole-mouth indices such as the Silness–Löe plaque index (PI) (24), the Attin plaque index (25), the Turesky modification of the Quigley–Hein index (TQHI) (26, 27), and the percentage plaque index (PPI) were used (18). To evaluate interproximal plaque accumulation, studies employed indices like percentage interproximal plaque index (PIPI) (18) and the modified approximal plaque index (MAPI) (28). Several studies also used orthodontic-specific indices, including the bonded bracket plaque index (BBPI) (29) and the plaque index around brackets (PIB) (30, 31), which better reflect plaque accumulation in patients with fixed appliances.

Gingival health was commonly assessed using the gingival index (GI), based on either the World Health Organization (WHO) or Silness–Löe criteria (32), alongside bleeding-related measures such as the gingival bleeding index (GBI) (33) and bleeding on probing (BOP) (23). Periodontal conditions were further evaluated using probing pocket depth (PPD) and clinical attachment level (CAL) (23).

In addition to periodontal indices, one study also assessed dental caries outcomes using the decayed, missing, and filled teeth/surface indices (DMFT/DMFS), biochemical markers and toothbrushing duration (20). In terms of patient-reported outcomes, one trial assessed user experience with interdental cleaning aids using a visual analogue scale (VAS) (19).

#### 3.2.5 Assessment of risk of bias

**Figure 2:**
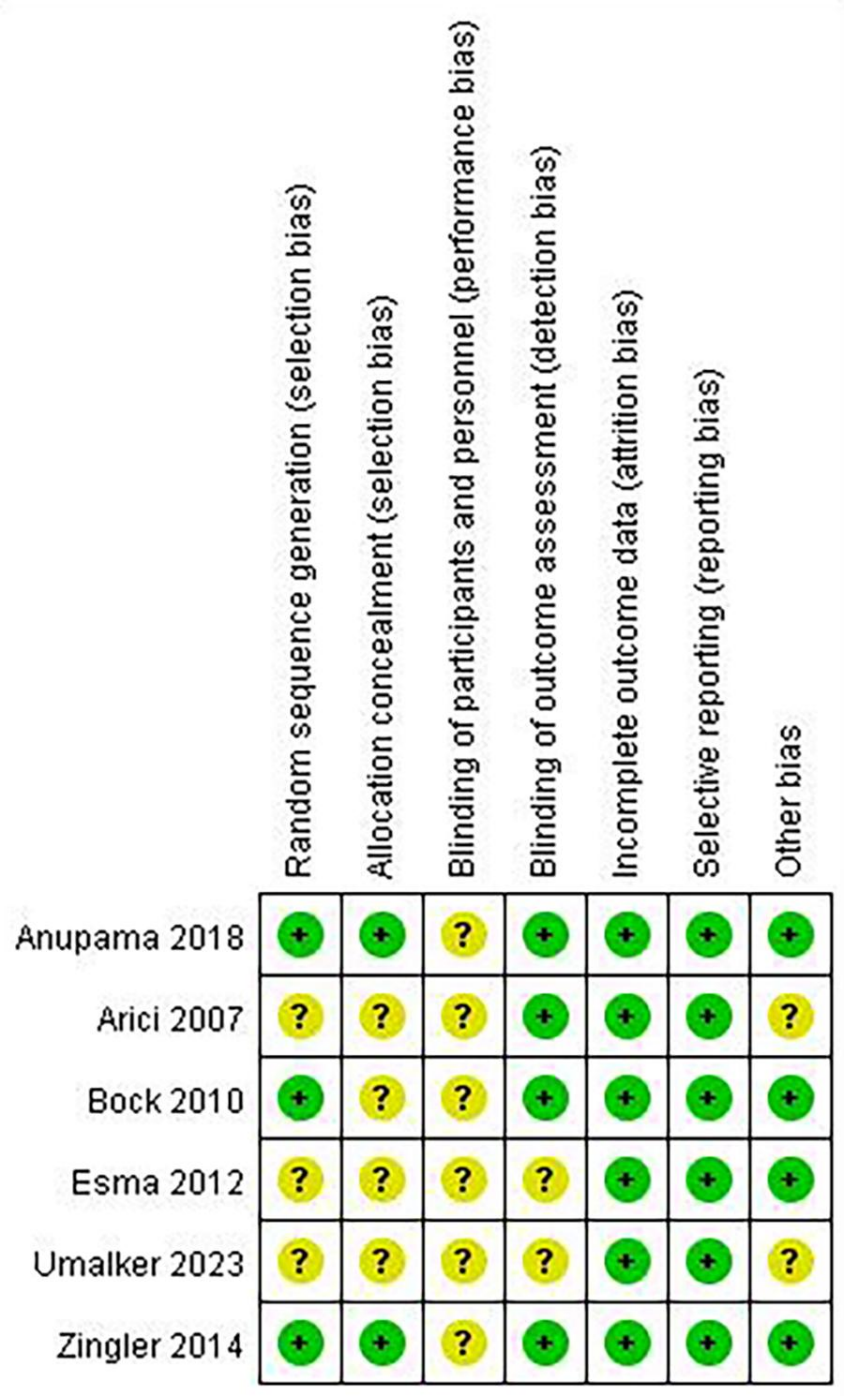
Risk of bias summary.

Across the seven Cochrane domains, no study was judged at high risk in any domain (Figure 2). Random sequence generation and allocation concealment were adequately reported in three trials (19, 20, 22) and unclear in the remaining three (18, 21, 23). Blinding of participants and personnel (performance bias) was unclear in all six studies, reflecting the practical impossibility of masking users to interdental cleaning devices. Blinding of outcome assessment (detection bias) was low risk in five trials and unclear in one (21). Incomplete outcome data (attrition bias) and selective reporting were consistently rated low risk across all studies. Two studies did not report conflicts of interest and were rated as unclear risk in the domain of other bias (18, 21).

### 3.3 Main outcomes

Given the small number of included studies and substantial between-study heterogeneity, we synthesized the evidence using a SWiM approach rather than conducting a meta-analysis. Where data were available, effect directions, MD, and 95% CI were summarized in Tables 3, 4.

**Table 3:** Summary of plaque and gingival outcomes for (toothbrushing + IDB) versus toothbrushing at week 4.

Author/Year	Intervention	Plaque outcome	Effect	MD (95% CI)	Gingival outcome	Effect	MD (95% CI)
Arici et al. (18)	OT + IDB vs. OT	PPI	**↑**	−5.04 (−8.07 to −2.01)	GI	**↑**	−0.38 (−0.52 to −0.24)
		PIPI	**↑**	−10.4 (−15.68 to −5.12)			
Anupama et al. (22)	OT + IDB vs. OT	BBPI	**↑**	−1.24 (−1.42 to −1.06)	GI	**↑**	−0.74 (−0.91 to −0.57)
					GBI	**↑**	−16.28 (−19.23 to −13.32)
Zingler et al. (20)	MTB + IDB + SS vs. MTB + SS	MAPI	**→**	Not applicable*	Not reported		
		TQHI	**→**				
		PIB	**→**				

↑: Favorable effect in IDB group; →: No significant between-group difference.

* Median and IQR reported in the original study; only the direction of effect is shown.

**Table 4:** Summary of plaque and gingival outcomes for IDB versus other interdental adjuncts.

Author/Year	Intervention	Outcome	Effect	MD (95% CI)	Follow-up
Bock et al. (19)	MTB + IDB vs. MTT + MTB	PI	**→**	Not reported	24 weeks
		VAS	**↑**		24 weeks
Umalkar et al. (21)	TB + IDB vs. floss + TB	PI	**↑**	−0.696 (−0.91 to −0.48)	3 months
		GI	**↑**	−0.78 (−0.91 to −0.65)	3 months
Esma et al. (23)	MTB + IDB vs. MTB + OI	PI	**↓**	Not available*	8 weeks
		GI	**↓**		8 weeks
		BOP	**↓**		8 weeks
		CAL	→		8 weeks
		PPD	→		8 weeks
		IL-10, MMP-1	→		8 weeks
		IL-1B, MMP-8	↓		8 weeks

↑: favorable effect in IDB group; →: no significant between-group difference; ↓: worse effect in IDB group;.

MTT, monotufted head toothbrush; OI, oral irrigator.

* Median and IQR reported in the original study; only the direction of effect is shown.

#### 3.3.1 Toothbrushing plus interdental brush vs. toothbrushing

##### 3.3.1.1 Plaque outcome

This comparison included three studies (unclear risk of bias) (18, 20, 22). Two studies evaluated the effect of (OT + IDB) vs. OT alone on plaque reduction and found significantly lower plaque levels in the IDB group (18, 22). One study compared (MTB + IDB) with MTB, with sealant used as a co-intervention, and reported no significant difference in MAPI, TQHI and PIB at the 8-week follow-up (20). These results suggest that toothbrush type may be a major source of heterogeneity. To enhance comparability, outcomes at the 4-week follow-up were presented in Table 2.

##### 3.3.1.2 Gingival outcome

Only two studies were included in this comparison (unclear risk of bias), both evaluating the difference in GI between (OT + IDB) and OT alone (18, 22). At the 4-week follow-up, both studies demonstrated that the use of IDB resulted in a reduction in GI. The 95% CIs in both studies did not cross zero, indicating a statistically significant effect. Additionally, IDB use was associated with lower GBI (22).

##### 3.3.1.3 Behavioral and caries outcomes

In addition to plaque and gingival outcomes, one study also assessed toothbrushing duration and caries-related measures (unclear risk of bias). The (MTB + IDB) group demonstrated a significantly longer average brushing time (∼ 255 s), although with greater individual variability. However, a declining trend in brushing time over the 12-week period was noted in both the MTB and (MTB + IDB) groups. DMFT and DMFS remained largely stable throughout the follow-up, with no significant differences observed between groups.

#### 3.3.2 Interdental brush vs. other interdental adjuncts

Several intervention comparisons were each represented by a single study, with their effect directions and effect sizes summarized in Table 3. One study with a split-mouth crossover design compared (MTB + IDB) to (monotufted head toothbrush + MTB) over 24 weeks and found no difference in PI but reported greater user satisfaction (VAS) with IDB (19). Another study compared (toothbrushing + IDB) with (floss + toothbrushing) over a 3-month follow-up and reported improvements favoring IDB for both plaque and gingival outcomes, with mean differences indicating lower PI (MD −0.696, 95% CI −0.91 to −0.48) and GI (MD −0.78, 95% CI −0.91 to −0.65) (21). One 8-week study compared (MTB + IDB) with (MTB + oral irrigator) and found effects favoring the oral irrigator for PI, GI, and BOP, suggesting less benefit from IDB in this context (23). No significant differences were observed between groups for CAL, PPD, IL−10, or MMP-1. Given the single-study nature and contextual heterogeneity, these findings should be interpreted with caution.

### 3.4 Certainty of evidence

Using the GRADE framework, the overall certainty of evidence was assessed as low across all comparisons (Table 5). This was primarily due to concerns about imprecision and unclear risk of bias, as most outcomes were derived from single-study comparisons with small sample sizes. For the comparison of (OT + IDB) vs. OT, low-certainty evidence from individual studies favored IDB across several plaque and gingival outcomes. Similar benefits were observed in (TB + IDB) vs. (TB + floss), though also based on single-study evidence.

**Table 5:** GRADE assessment of the certainty of the evidence.

Outcome	Number of participants and studies	Effect	Quality of evidence	Reasons for downgrading
(OT + IDB) vs. OT				
PIPI	30 (1 study)	In favor of IDB	low ⬤⬤◯◯	unclear risk of bias, imprecision
PPI	30 (1 study)	In favor of IDB	low ⬤⬤◯◯	unclear risk of bias, imprecision
BBPI	60 (1 study)	In favor of IDB	low ⬤⬤◯◯	unclear risk of bias, imprecision
GI	90 (2 studies)	In favor of IDB	low ⬤⬤◯◯	unclear risk of bias, imprecision
GBI	60 (1 study)	In favor of IDB	low ⬤⬤◯◯	unclear risk of bias, imprecision
(MTB + IDB + SS) vs. (MTB + SS)				
MAPI	59 (1 study)	No difference	low ⬤⬤◯◯	unclear risk of bias, imprecision
TQHI	59 (1 study)	No difference	low ⬤⬤◯◯	unclear risk of bias, imprecision
PIB	59 (1 study)	No difference	low ⬤⬤◯◯	unclear risk of bias, imprecision
(MTB + IDB) vs. (MTT + MTB)				
PI	104 (1 study)	No difference	low ⬤⬤◯◯	unclear risk of bias, imprecision
VAS	104 (1 study)	In favor of IDB	low ⬤⬤◯◯	unclear risk of bias, imprecision
(TB + IDB) vs. (TB + floss)				
PI	100 (1 study)	In favor of IDB	low ⬤⬤◯◯	unclear risk of bias, imprecision
GI	100 (1 study)	In favor of IDB	low ⬤⬤◯◯	unclear risk of bias, imprecision
(MTB + IDB) vs. (MTB + OI)				
PI	30 (1 study)	Against IDB	low ⬤⬤◯◯	unclear risk of bias, imprecision
GI	30 (1 study)	Against IDB	low ⬤⬤◯◯	unclear risk of bias, imprecision
BOP	30 (1 study)	Against IDB	low ⬤⬤◯◯	unclear risk of bias, imprecision
CAL	30 (1 study)	No difference	low ⬤⬤◯◯	unclear risk of bias, imprecision
PPD	30 (1 study)	No difference	low ⬤⬤◯◯	unclear risk of bias, imprecision
IL-10, MMP-1	30 (1 study)	No difference	low ⬤⬤◯◯	unclear risk of bias, imprecision
IL-1B, MMP-8	30 (1 study)	Against IDB	low ⬤⬤◯◯	unclear risk of bias, imprecision

In contrast, for the comparison of (MTB + IDB + SS) vs. (MTB + SS), evidence from one study indicated no significant differences in plaque outcomes, including MAPI, TQHI, and PIB (20). Although the brushing regimen was more time-intensive in the IDB group, no clinical superiority was observed. All outcomes in this comparison were rated as low-certainty due to unclear risk of bias and imprecision. One study comparing (MTB + IDB) with (MTB + OI) showed effects against IDB across all reported outcomes (23), while another comparing (MTB + IDB) with (MTT + MTB) found no difference in plaque levels but a slight improvement in patient satisfaction (19). Due to reliance on single trials with small sample sizes and limited reporting quality, all comparisons were downgraded, and findings should be interpreted with caution.

## 4 Discussion

Maintaining adequate oral hygiene is particularly challenging for patients undergoing fixed orthodontic treatment due to the increased risk of plaque accumulation around brackets, bands, and wires, which can lead to gingival inflammation, enamel decalcification, and caries development. Effective adjunctive cleaning methods, particularly for interproximal areas, are thus essential to support periodontal health during orthodontic therapy. This synthesis of randomized controlled trials evaluated the clinical efficacy of interdental brushes as adjuncts to toothbrushing in orthodontic patients.

In studies employing orthodontic toothbrushes as the base regimen, the combination of (OT + IDB) consistently demonstrated superior performance across multiple plaque and gingival indices, with statistically significant mean differences and effect directions favoring the intervention. These results align with previous findings in non-orthodontic populations, where IDB has been identified as the most effective interproximal cleaning tool for reducing gingival inflammation (13). Notably, in one study, IDB was preferred over monotufted brushes due to superior user experience (19), further reinforcing its potential for real-world application.

However, the evidence was not uniform across all studies. One trial that combined manual toothbrushing and IDB with a sealant co-intervention failed to show significant differences in plaque or gingival scores when compared with MTB alone (20). Participants in the IDB group spent significantly more time on toothbrushing, although this increase in brushing time did not correspond to superior clinical outcomes. Similarly, another trial comparing IDB with oral irrigators reported inferior outcomes for IDB across plaque, gingival index, and bleeding on probing (23). These discrepancies may reflect important clinical and methodological heterogeneity, including differences in toothbrush type, study design, adjunctive co-interventions, brushing instructions and supervision, and participants' baseline oral hygiene status.

These results contribute to the growing consensus that interdental cleaning is essential for comprehensive plaque control in orthodontic patients, particularly in hard-to-reach interproximal areas. Our findings refine this understanding by highlighting that the clinical benefit of IDB appears to be contingent on the type of toothbrush used. Orthodontic toothbrushes, with V-shaped bristle designs, may enhance the mechanical compatibility and synergistic action of IDB around brackets and wires. In contrast, when used with standard manual toothbrushes, the marginal utility of IDB may be limited due to user-dependent variability or technique sensitivity.

Another source of variation lies in the outcome measures. Although validated indices were used, studies differed in scale selection and follow-up duration. The generally short observation periods may limit detection of sustained effects, especially given the Hawthorne effect—where participants temporarily improve hygiene due to being observed. To reduce this bias, a run-in phase with baseline hygiene education has been recommended (34), helping to stabilize behavior before outcome assessment. Only one trial included biochemical markers (23), and just one captured patient-reported outcomes like ease-of-use and discomfort (19). While clinical indices are important, user experience plays a key role in adherence and real-world effectiveness especially among adolescents. The reported preference for IDB over monotufted brushes highlights its potential to improve compliance, which warrants further investigation through longer-term and mixed-method studies.

Importantly, several included trials showed clinically relevant baseline imbalances or pre-intervention procedures that may also confound effect estimates. For example, one trial that compared interdental brushes with floss reported a marked age distribution difference between arms (interdental-brush group skewed younger than the floss group), which could influence both baseline oral hygiene and adherence patterns and therefore the observed treatment effect. In addition, one study enrolled participants with high baseline plaque levels, while others performed professional prophylaxis immediately before randomization. Both scenarios may modify absolute effect sizes and hinder between-study comparability. These study-level differences should be considered when interpreting pooled directions of effect and highlight the need for future trials to ensure balanced randomization and transparent reporting of baseline characteristics and pre-trial prophylaxis.

In addition to variation in toothbrush type, outcome measures and baseline characteristics, differences in study design are likely contributors. Two studies adopted crossover or split-mouth designs, which are efficient for reducing inter-individual variability but are inherently prone to carryover effects if washout periods are insufficient or if learning effects influence participants' technique over time.

The overall quality of evidence, as assessed by GRADE, was low across all comparisons. This is largely attributable to small sample sizes, reliance on single-study estimates, and imprecision in outcome reporting. Although several studies were rated as having unclear risk of bias in performance domains, this largely reflects the inherent difficulty of blinding participants in oral hygiene intervention trials, where device-related differences are obvious to users. Consequently, this limitation should be interpreted as a methodological constraint rather than a flaw in trial execution. Nonetheless, inadequate reporting of randomization procedures, heterogeneity in study protocols, and absence of assessor blinding in some trials further limit confidence in the findings.

Several methodological shortcomings warrant caution. First, nearly all comparisons were based on single trials with small sample sizes and limited geographic diversity, undermining the generalizability of findings. Second, most trials had short follow-up durations, precluding the assessment of long-term outcomes such as enamel decalcification, caries incidence, or sustained behavioral adherence. Third, despite using validated periodontal indices, there was heterogeneity in outcome selection, measurement timing, and reporting methods. Blinding was infeasible in all studies, given the visible nature of interventions. However, this should be viewed as a structural limitation rather than a flaw. Additionally, one of the included studies was a preprint (23). Grey literature was not comprehensively searched, and results could not be obtained from 12 potentially eligible studies. These factors may have introduced publication bias.

This review provides a nuanced synthesis that clarifies when and how IDBs might be most beneficial. While traditional guidelines recommend interdental cleaning for all orthodontic patients, our results suggest tailoring IDB recommendations based on toothbrush compatibility, patient preference, and clinical context may improve outcomes. We extend the current view by emphasizing the role of device synergy, patient experience, and behavioral reinforcement as equally critical determinants of success, beyond clinical efficacy alone. Future research should move beyond short-term clinical endpoints and explore:

- The longitudinal impact of IDB use on enamel demineralization, caries prevention, and microbiota shifts;
- The behavioral adherence mechanisms associated with IDB use, possibly mediated by user preference or ease-of-use;
- The effect modification by toothbrush design, especially under supervised vs. unsupervised brushing conditions;
- The interaction between patient age, dexterity, and device usability, particularly among adolescents.

## 5 Conclusion

Low-certainty evidence from a limited number of heterogeneous trials provides only tentative indications that interdental brushes may help improve gingival health and plaque control in orthodontic patients—particularly when used with orthodontic toothbrushes. High-quality, multicenter trials with standardized protocols and longer follow-up are needed to guide clinical recommendations.

## Data Availability

The original contributions presented in the study are included in the article/Supplementary Material, further inquiries can be directed to the corresponding author.
