# Supplementary material for: Interdental brushes for improving oral hygiene in patients with fixed orthodontic appliances: a systematic review
Source: Front Oral Health. 2026 May 4;7:1707988. doi: 10.3389/froh.2026.1707988 (PMC13180904; doi:10.3389/froh.2026.1707988)
Supplement: Supplementary file 1 [file Table1.docx]

Supplementary Material

Interdental brushes for improving oral hygiene in patients with fixed orthodontic appliances: a systematic review

# Supplementary Table S1. Search strategies

PubMed

|  | Items |
| --- | --- |
| #1 | ((((((((Orthodontic Appliances, Fixed[MeSH Terms]) OR (fixed orthodontic appliance[Title/Abstract])) OR (fixed orthodontic appliances[Title/Abstract])) OR (fixed appliance[Title/Abstract])) OR (fixed appliances[Title/Abstract])) OR (orthodontic brackets[Title/Abstract])) OR (orthodontic wires[Title/Abstract])) OR (braces[Title/Abstract])) OR (orthodontic[Title/Abstract]) |
| #2 | ((((interdental brush[Title/Abstract]) OR (interproximal brush[Title/Abstract])) OR (proxabrush[Title/Abstract])) OR (interdental[Title/Abstract])) OR (interproximal[Title/Abstract]) |
| #3 | ((((Randomized Controlled Trial[Publication Type]) OR (randomized controlled trial[Title/Abstract])) OR (randomised controlled trial[Title/Abstract])) OR (RCT[Title/Abstract])) OR (clinical trial[Title/Abstract]) |
| #4 | #1 AND #2 AND #3 |

Embase

|  | Items |
| --- | --- |
| #1 | ('interdental brush':ti,ab,kw OR 'interproximal brush':ti,ab,kw OR proxabrush:ti,ab,kw OR interdental:ti,ab,kw OR interproximal) AND ('fixed orthodontic appliance':ti,ab,kw OR 'fixed appliance':ti,ab,kw OR braces:ti,ab,kw OR 'orthodontic brackets':ti,ab,kw OR 'orthodontic'/exp OR orthodontic) AND 'clinical trial'/it |

Cochrane

|  | Items |
| --- | --- |
| #1 | (interdental brush OR interproximal brush OR proxabrush OR interdental OR interproximal):ti,ab,kw AND (fixed orthodontic appliance OR fixed appliance OR braces OR orthodontic brackets OR orthodontic):ti,ab,kw |

Web of Science

|  | Items |
| --- | --- |
| #1 | interdental brush OR interproximal brush OR proxabrush OR interdental OR interproximal(Topic) AND fixed orthodontic appliance OR fixed appliance OR braces OR orthodontic brackets OR orthodontic(Topic) AND clinical trial OR randomized controlled trial(Topic) |

Scopus

|  | Items |
| --- | --- |
| #1 | TITLE-ABS-KEY (interdental brush OR interproximal brush OR proxabrush)  AND TITLE-ABS-KEY (fixed orthodontic appliance OR fixed appliance OR braces OR orthodontic brackets) AND TITLE-ABS-KEY (clinical trial OR randomized controlled trial) |

# Supplementary Table S2. Reasons for full-text articles exclusion

| Title | Reasons for exclusion |
| --- | --- |
| Comparison of the cleaning effectiveness of the Philips Sonicare AirFloss Ultra (HX 8331/51) and the miradent I-Prox® P bursh used for the prevention of gingivitis and caries during orthodontic treatments with fixed appliances | The comparison was between an oral irrigator and a monotufted toothbrush, and was unrelated to IDB. |
| Effectiveness of three different types of electric toothbrushes compared with a manual technique in orthodontic patients | The intervention was unrelated to IDB. |
| Effectiveness of Different Types of Toothbrushes on Periodontal Health in Orthodontic Patients | The intervention was unrelated to IDB. |
| Single-blind randomized clinical trial on the efficacy of an interdental cleaning device in orthodontic patients | The interdental cleaning device was unrelated to IDB. |
| Evaluation of interdental cleaning in orthodontic patients in Hong Kong | The intervention was unrelated to IDB. |
| Randomized multicenter study on the plaque removal efficacy of 2 interdental brushes around the base of orthodontic brackets | Toothbrushing was not included as part of the intervention. |
| Influence of three types of toothbrushes on enamel decalcification in orthodontic patients: a comparative study | Toothbrushing was not included in the IDB group. |
